# Lignin-Derived Activated Carbon as Electrode Material for High-Performance Supercapacitor

**DOI:** 10.3390/molecules30010089

**Published:** 2024-12-29

**Authors:** Chenghao Pan, Yongfeng Ji, Suxia Ren, Tingzhou Lei, Lili Dong

**Affiliations:** 1 School of Environmental Science and Engineering, Changzhou University, Changzhou 213164, China; s22030857045@smail.cczu.edu.cn; 2 Institute of Urban & Rural Mining, Changzhou University, Changzhou 213164, China; 13222666378@163.com (Y.J.); rensuxia@cczu.edu.cn (S.R.)

**Keywords:** lignin, activated carbon, KOH activation, supercapacitors

## Abstract

Utilizing lignin-derived activated carbon in supercapacitors has emerged as a promising approach to alleviating environmental pollution and promoting the high-value utilization of byproducts in the papermaking industry. In this study, activated carbons (LACs) were prepared using a simple one-step KOH activation approach and by employing enzymatic hydrolysis lignin (EHL). The impact of the KOH activation parameters on the microstructure and capacitive performance of the LACs was investigated by varying the KOH/EHL ratio and activation temperature. The optimized sample LAC_800-4_ showed an interconnected porous structure with a high surface area of 2285 m^2^/g, abundant micropores, and a small number of mesopores, which makes it a suitable electrode material for supercapacitors. The sample LAC_800-4_ demonstrated a high specific capacitance of 291.3 F/g in a three-electrode system. Under a symmetrical supercapacitor electrode system, the specific capacitance of the LAC_800-4_ electrode reached 186.8 F/g at 0.5 A/g. After 10,000 cycles at 20 A/g, the capacitance retention rate remained at 96.1%. The symmetrical supercapacitor also demonstrated a superior energy density of 6.5 Wh/kg. This work provides valuable insights into the transformation of low-value natural biomass derivatives into environmentally friendly, high-performing supercapacitor electrode materials.

## 1. Introduction

In recent years, the use of carbon-based supercapacitors in energy storage and conversion devices has garnered significant attention due to their high energy density, rapid charge and discharge rates, and superior safety performance [1,2]. Based on the formation mechanism of double-layer capacitance, the carbon material electrode should contain abundant micropores to provide a high surface area, enough active sites for electron storage, and an appropriate amount of mesopores and macropores to supply fast transmission and diffusion channels for the electrolyte [3,4]. Therefore, preparing activated carbon materials that meet the above requirements has gained considerable attention. 

In recent years, biomass has been recognized as an ideal precursor for activated carbon due to its renewability, chemical stability, low cost, and unique structure. Wu et al. [5] prepared honeycomb-like carbon foam from wheat flour via one-step carbonization. The unique structure provided the carbon foam with a significant capacitive performance, with a high specific capacitance of 473 F/g at 0.5 A/g and a high energy density of 29.3 63.5 Wh/kg. Chen et al. [6] used rice husk as a raw material to prepare hierarchical porous carbon. The contribution of different rice husk components, including cellulose, hemicellulose, lignin, and SiO_2_, to the formation of pores was investigated. Lignin mainly contributes to the formation of mesopores and micropores, which is crucial in obtaining a high specific capacitance. The optimized sample showed a specific capacitance of 51.4 F/g at 0.5 A/g in the symmetric supercapacitor, while its capacitance retention was as high as 96.2 % at 20 A/g. Studies have shown that biomass-derived carbon materials could be effectively employed in supercapacitors. Among the numerous biomass precursors developed, lignin has attracted significant attention due to its high carbon content, numerous self-doping heteroatoms, rigid chemical structure, and large and cheap output.

Lignin is the most abundant aromatic polymer in the world. Approximately 70 million tons of lignin are produced from pulping byproducts annually, but almost 98% of that lignin is burned as low-quality fuel or discarded directly as waste [7,8]. The conversion of lignin into carbon-based electrode materials not only alleviates environmental pollution but also realizes the high-value utilization of lignin. In recent years, some methods for the preparation of lignin-based carbon materials for use in supercapacitors have been developed. Zhang et al. [9] prepared carbon materials from lignin with and without KOH. The carbon material (LC) obtained without KOH showed a large texture with a small surface area, while the hierarchical porous carbon (LHPC) prepared with KOH showed a high surface area of 907 m^2^/g. Benefiting from the hierarchical porous structure, the LHPC sample presented a superior captive performance, with a specific capacitance of 165.0 F/g in 1M H_2_SO_4_ at 0.05 A/g. Shi et al. [10] fabricated porous carbon from enzymatically hydrolyzed lignin and urea through a KOH activation procedure. The influences of activation temperatures and elemental doping on the microstructure and capacitive performance of the synthesized ONS-HPC materials were investigated and the ONS-HPC-600 electrode material presented the best electrochemical performance. It can be concluded that the activation parameters strongly influence the microstructure and capacitive performance of carbon materials. However, systematic discussions regarding the influence of the activation parameters on the pore structure and electrochemical performance of lignin-derived activated carbon have rarely been reported.

In this study, activated carbons (LACs) were prepared from enzymatic hydrolysis lignin and KOH using a facile one-step method. The lignin-derived activated carbon was characterized to analyze the mechanism implicated in the formation of the porous structure of LACs. The intrinsic regular relationship between the structure and electrochemical performance of LACs was further discussed. This work will provide a valuable reference for the fabrication of lignin-derived carbon electrodes for supercapacitors.

## 2. Results and Discussion

**Figure 1:**
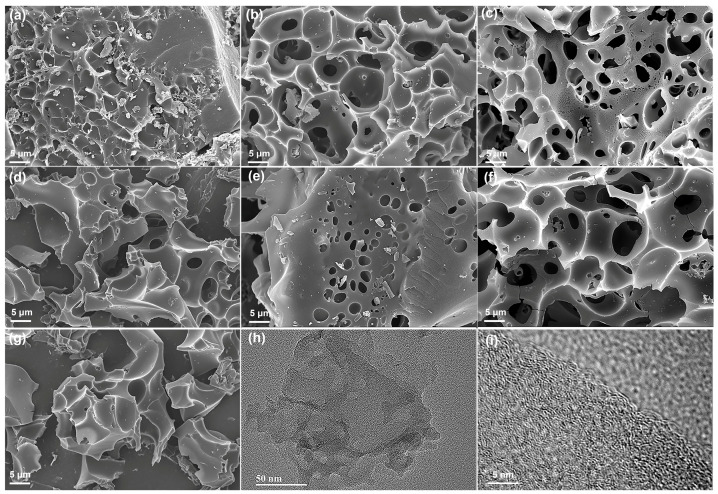
SEM images of (**a**) LAC_800-2_, (**b**) LAC_800-3_, (**c**) LAC_800-4_, (**d**) LAC_800-5_, (**e**) LAC_600-4_, (**f**) LAC_700-4_, and (**g**) LAC_900-4_; (**h**) TEM and (**i**) HRTEM images of LAC_800-4_.

Scanning electron microscopy (SEM) was used to characterize the morphology and microstructure of the as-prepared samples. As shown in Figure 1a, LAC_800-2_ exhibited a bulk structure, in which only the surface was etched. This may be attributed to the low quantity of the KOH added, which contributed to the low etching degree and relatively complete carbon skeleton. As the KOH/EHL mass ratio increased, the LAC_800-3_ and LAC_800-4_ formed a well-developed porous structure. Generally speaking, an appropriate porous structure should contain a large number of micropores to provide enough active sites for ion storage, a certain amount of mesopores to provide short and convenient ion transfer channels, and large pores to provide buffer layers for electrolyte ions [11]. Thus, LAC_800-3_ and LAC_800-4_, which have interconnected porous structures, may exhibit a significant capacity for electrochemical energy storage due to the improved electron and ion transport efficiency. However, the carbon skeleton was over-etched due to the excessive use of KOH, as indicated in Figure 1d [12]. The activation temperature also has a strong impact on the porous structure of carbon materials. The LAC_600-4_ sample showed few pores on its surface due to the low activation temperature used, namely 600 °C; this hindered the process of KOH activation. As the activation temperature increased to 700 and 800 °C, a porous structure was achieved due to the suitable etching of KOH (Figure 1c,f). However, using an overly high activation temperature of 900 °C led to the collapse of LAC_900-4_’s structure (Figure 1g). The TEM image shows that LAC_800-4_ had a rough surface with numerous pores. The HRTEM image indicated the typical porous amorphous structure of LAC_800-4_ [13].

**Figure 2:**
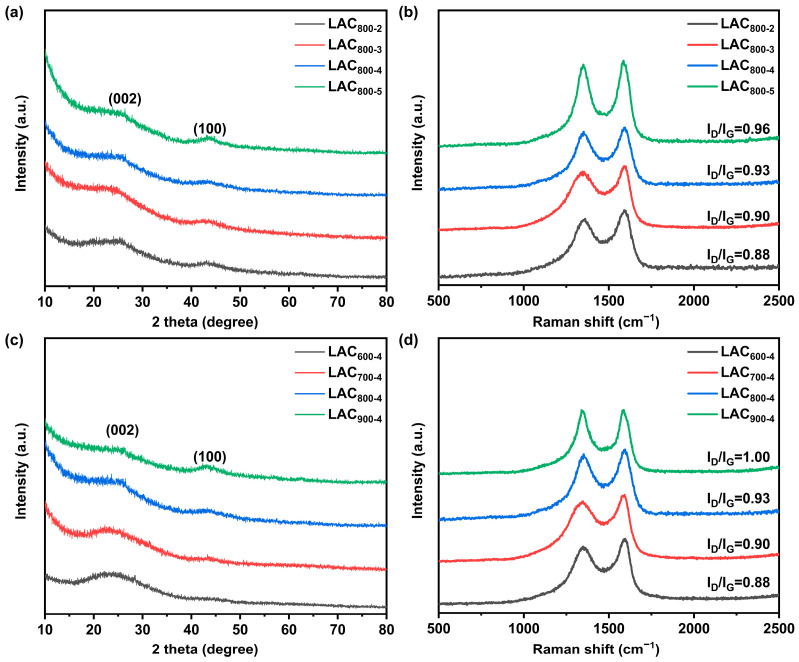
XRD patterns (**a**) and Raman spectra (**b**) of LAC_800-2_, LAC_800-3_, LAC_800-4_, and LAC_800-5_; XRD patterns (**c**) and Raman spectra (**d**) of LAC_600-4_, LAC_700-4_, LAC_800-4_, and LAC_900-4_.

To study the crystal structure of the samples, XRD and Raman tests were performed, as shown in Figure 2. The samples prepared with various KOH contents presented two typical diffraction peaks at 25 and 43 degrees, which were ascribed to the (002) and (100) crystal planes of carbon (Figure 2a) [14]. The broad and low peaks indicate the low crystallinity and typical amorphous carbon structure of the LAC_800-2_, LAC_800-3_, LAC_800-4_, and LAC_800-5_ samples [15]. As the KOH content increased, the (002) crystal plane showed a gradual reduction in diffraction peak intensity. This suggests that the structure of LAC_s_ was etched by KOH; this reduced its regularity, creating visible pores or faults and lowering the degree of graphitization [16]. The Raman spectra degree was further studied to investigate the graphitization of the LAC_800-2_, LAC_800-3_, LAC_800-4_, and LAC_800-5_ samples. According to Figure 2b, two peaks at 1330 and 1585 cm^−1^ appeared in all the Raman spectra, which were ascribed to the defect-induced D-band and ideal graphite-derived G band, respectively. The degree of irregular arrangement in the LACs was estimated using the I_D_/I_G_ ratio, which was calculated via Gaussian function fitting. The I_D_/I_G_ values of LAC_800-2_, LAC_800-3_, LAC_800-4,_ and LAC_800-5_ were calculated to be 0.88, 0.90, 0.93, and 0.96, respectively. These results suggest that the degree of graphitization decreases as the amount of KOH activator increases. The XRD patterns of LAC_600-4_, LAC_700-4_, LAC_800-4,_ and LAC_900-4_ (Figure 2c) all showed relatively wide diffraction peaks, indicating that the samples possessed amorphous structures [17]. The Raman spectra further confirmed this finding. With the increase in the activation temperature, the ratio of I_D_/I_G_ gradually increased, indicating that the degree of graphitization decreased. This is mainly because the graphitization of carbon materials occurs at temperatures above 1000 °C. When the activation temperature is 600–900 °C and the mass ratio of KOH to EHL is high, the activation of KOH plays a dominant role, leading to an increase in the degree of disorder in the samples [18].

**Figure 3:**
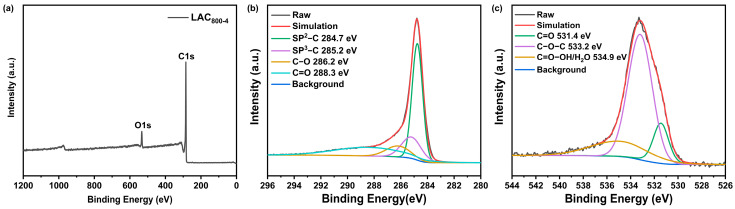
Full XPS spectrum of LAC_800-4_ (**a**); peak fitting spectra of C 1s (**b**) and O 1s (**c**) of LAC_800-4_.

XPS spectra were selected to further determine the chemical composition and valence states of LAC_800-4_. As shown in Figure 3a, the survey XPS spectrum of LAC_800-4_ revealed the presence of C and O, which were the major elements. Four sub-peaks at 284.7 eV, 285.6 eV, 286.5 eV, and 288.9 eV were fitted in the C1s spectrum, and these were attributed to the sp^2^ C=C, sp^3^ C-C, C-O, and C=O bands, respectively [19]. In the high-resolution O 1s spectrum, there were three characteristic peaks at 531.4, 533.3, and 534.9 eV, which were attributed to C=O, C-O-C, and C=O-OH or water, respectively [20]. The presence of the C=O group promoted the wettability of the LAC_800-4_ surface, which could reduce the charge impedance of the supercapacitor. The oxygen-containing functional groups could also serve as the active sites to enhance the capacitive performance via a reversible redox reaction [21].

**Figure 4:**
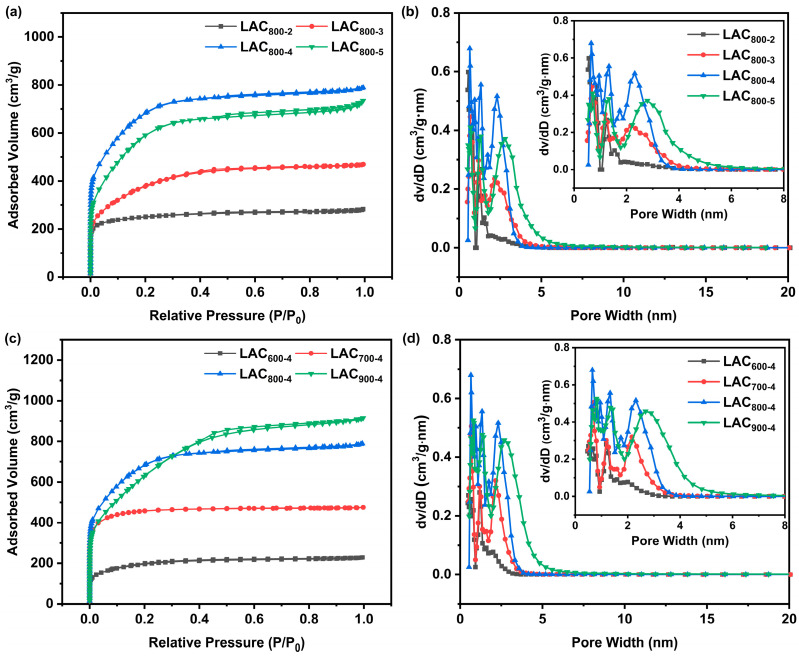
N_2_ adsorption–desorption curves (**a**) and pore size distribution curves (**b**) of LAC_800-2_, LAC_800-3_, LAC_800-4_, and LAC_800-5_; N_2_ adsorption–desorption curves (**c**) and pore size distribution curves (**d**) of LAC_600-4_, LAC_700-4_, LAC_800-4_, and LAC_900-4_.

N_2_ adsorption–desorption analysis was used to further investigate the microstructures of the LACs, as illustrated in Figure 4. All the LACs presented I-type isothermal curves and H4-type hysteresis rings. The isothermal curve of LAC_800-2_ exhibited a significant increase in P/P_0_ < 0.02, indicating the existence of a large number of micropores [21]. In addition to the sharp rise in P/P_0_ < 0.01, a rising stage in the range of 0.02 < P/P_0_ < 0.4 appeared for the LAC_800-3_, LAC_800-4_, and LAC_800-5_ samples. As the KOH/EHL mass ratios increased, the H4-type hysteresis ring became larger, indicating that the number of mesopores increased. The slight rise in the range of 0.9 < P/P_0_ < 1.0 reveals the presence of some macropores [22]. The N_2_ adsorption–desorption isotherm of samples under different activation temperatures exhibited similar characteristics (Figure 4c). In particular, the LAC_900-4_ sample exhibited a larger hysteresis loop and wider rising stage, suggesting an increased percentage of mesopores. These findings show that the LACs had a hierarchical porous structure, and the pore size distribution curves further confirmed these results (Figure 4b,d). All the LACs exhibited pore size distributions in the range of 0.6–2 nm and 2–6 nm, implying the existence of both micropores and mesopores.

It was found that the KOH/EHL mass ratio strongly influences the microstructures of LACs. A relatively low ratio of KOH/EHL (2:1) results in insufficient activation of the carbon skeleton, which only occurred on the surface of LAC_800-2_ (as presented in the SEM image). Along with the increase in the KOH/EHL mass ratio, the N_2_ adsorption capacity of LAC_800-3_ and LAC_800-4_ gradually increased. The LAC_800-4_ sample exhibited the highest surface area of 2285 m^2^/g. This is because the appropriate KOH activation led to a reasonable pore size distribution, with a large number of micropores and some mesopores [23]. However, the addition of excess KOH led to the collapse of the porous structure, thereby decreasing the surface area and widening the pore size distribution of LAC_800-5_. As illustrated in Figure 4c,d, the activation temperature is another factor that significantly affects the activation procedure. KOH activation is difficult to achieve at a low activation temperature of 600 °C, resulting in the low surface area observed in LAC_600-4_. The N_2_ adsorption capacity gradually increased as the activation temperature increased to 800 °C, implying that there was an increase in the surface area and number of micropores. The pore size distribution range also extended due to the more intensive reaction between the carbon skeleton and KOH at higher activation temperatures. However, strong KOH etching occurred at a high activation temperature of 900 °C, leading to a decrease in the number of micropores and an increase in the number of mesopores. In summary, the LACs prepared from lignin possessed an interconnected porous structure with an abundance of micropores and an appropriate number of mesopores and macropores. The dominating micropores in the electrode material provide a high surface area and abundant active sites for electron storage, while the mesopores and macropores enable fast transmission and diffusion channels for electrolytes [24].

**Figure 5:**
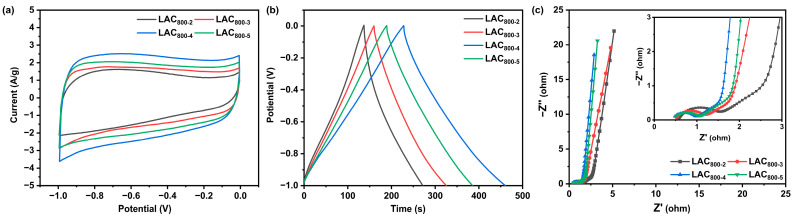
CV curves at 10 mV/s (**a**), GCD curves at 1 A/g (**b**), and Nyquist plots (**c**) of LAC_800-2_, LAC_800-3_, LAC_800-4_, and LAC_800-5_.

The electrochemical properties of the LAC_800-2_, LAC_800-3_, LAC_800-4_, and LAC_800-5_ samples were tested in a three-electrode system, as displayed in Figure 5. Under a scan rate of 10 mV/s, all the CV curves of LAC_s_ showed a quasi-rectangular shape, indicating their double-layer characteristics and capacity for a rapid electrode response [25]. Compared with the other samples, LAC_800-4_ presented the largest integrated area, showing the best capacitive performance. The GCD curves of LAC_S_ at 1 A/g were further examined, as illustrated in Figure 5b. All the GCD curves presented an approximate isosceles triangular shape, revealing the typical electric double-layer capacitance and reversibility of the electrode materials. As the KOH/EHL ratio increased, the specific capacitance of the LAC_S_ increased first and subsequently declined when the KOH/EHL ratio surpassed 3:1. The specific capacitances of LAC_800-2_, LAC_800-3_, LAC_800-4,_ and LAC_800-5_ are 168.8, 207.5, 291.3, and 246.3 F/g, respectively. The resistance of the prepared porous carbon electrodes is characterized by the electrochemical impedance spectroscopy (EIS) measurement, as illustrated in Figure 5c. The Nyquist plot curves of the four samples showed a semicircle at high frequencies and a straight line at low frequencies, corresponding to the interfacial charge–transfer resistance (R_ct_) and Warburg resistance (Z_w)_, respectively. The intercept of the curve on the real axis in the high-frequency region corresponds to the equivalent series resistance (R_s_) [26]. The LAC_800-4_ sample exhibited the smallest R_s_ and R_ct_ values of 0.42 and 0.53 Ω, demonstrating the superior charge transport properties. As discussed above, the LAC_800-2_ sample presented the smallest surface area, so it could not supply enough active sites for ion storage. As the KOH usage increased, a large number of micropores formed, which led to an increase in the surface area and specific capacitance. However, excessive KOH usage causes the collapse of the structure, which leads to a decreased specific capacitance. It can be concluded that the capacitive performance of lignin-based activated carbon can be adjusted by varying the KOH/EHL ratio.

**Figure 6:**
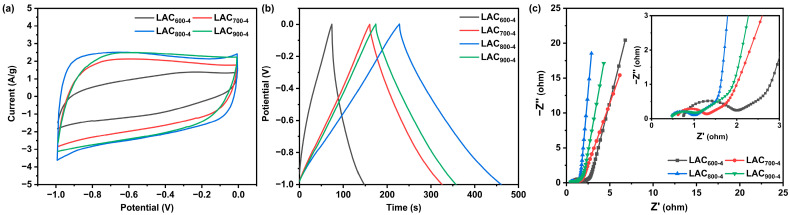
CV curves at 10 mV/s (**a**), GCD curves at 1 A/g (**b**), and Nyquist plots (**c**) of LAC_600-4_, LAC_700-4_, LAC_800-4_, and LAC_900-4_.

Figure 6 shows the electrochemical performance of the LAC_600-4_, LAC_700-4_, LAC_800-4_, and LAC_900-4_ samples under different activation temperatures (600–900 °C). It is known that a rectangular CV curve indicates the typical capacitive behavior of electrode materials. As shown in Figure 6a, the LAC_800-3_ sample presented a quasi-rectangular CV curve. However, the CV curves of other samples, especially LAC_600-4_, deviate from the rectangle. In particular, the LAC_800-4_ electrode exhibits the highest current density and largest closed area, implying the best supercapacitance performance. The GCD curves showed the typical electrical double-layer capacitance according to the near-isosceles triangle shape (Figure 6b) [27]. At a current density of 1 A/g, the highest specific capacitance, namely 291.3 F/g, was calculated for LAC_800-4_; meanwhile, LAC_600-4_, LAC_700-4_, and LAC_900-4_ exhibited specific capacitances of 92.5, 209.3, and 230.0 F/g, respectively. The activation temperature was also found to significantly influence the impedance (Figure 6c). The trends in R_s_ and R_ct_ were in the order of LAC_800-4_ < LAC_900-4_ < LAC_700-4_ < LAC_600-4_. The LAC_800-4_ sample exhibited the lowest R_s_ and R_ct_ values and the largest slope in the low-frequency region. At a low activation temperature of 600 °C, KOH was unable to activate the sample effectively, which resulted in a small number of micropores and a low specific capacitance [28]. However, a high activation temperature of 900 °C led to the over-etching of the carbon skeleton, thereby resulting in a decrease in the specific surface area. In addition, the enlarged micropores led to a decrease in the specific capacitance. Therefore, a moderate activation temperature (T = 800 °C) needs to be used alongside the ideal microporous carbon material, thereby maximizing the performance of supercapacitors.

**Figure 7:**
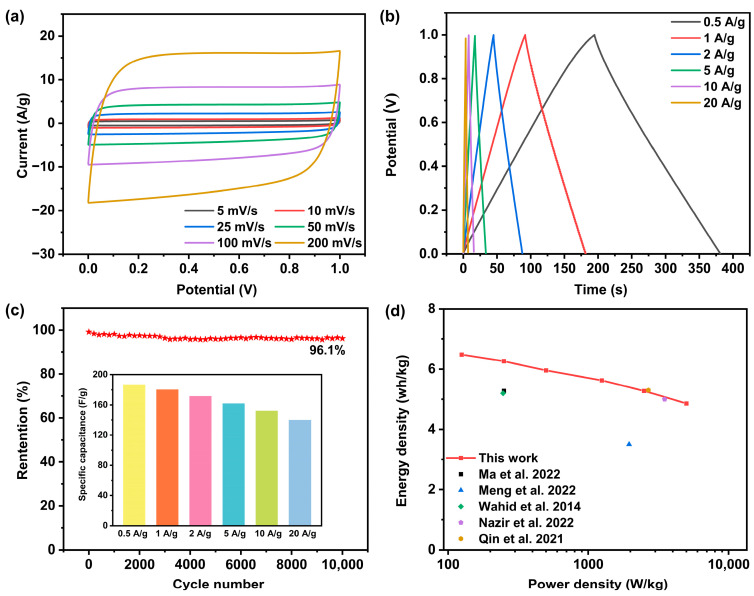
Electrochemical performance of the LAC_800-4_ electrode in a two-electrode system in 6 M KOH: (**a**) CV curves at various scan rates. (**b**) GCD curves at various current densities. (**c**) Cycling stability at 20 A/g for 10,000 cycles. (**d**) Ragone plots and comparison with other reported results. Refs. [20,23,30,31,32].

As the optimally prepared sample, the LAC_800-4_-based symmetrical supercapacitor was tested in a two-electrode system to evaluate the capacitive performance practically. Figure 7a shows the CV curves of the symmetric supercapacitor at different scan rates, from 10 to 200 mV/s. All the CV curves demonstrated a near-ideal rectangular shape. The CV curves showed no discernible distortion as the scanning rate increased, indicating a superior double-layer capacitive performance [29]. Figure 7b shows the GCD curves of the symmetric supercapacitor at different current densities. All the GCD curves demonstrated a symmetrical triangle shape, implying that the capacitance of the symmetric supercapacitor was reversible. The LAC_800-4_ electrode showed a specific capacitance of 186.8 F/g at 0.5 A/g and 140 F/g at 20 A/g, and the capacitance retention was calculated to be 74.9%. Furthermore, the durability of the symmetric supercapacitor was examined by performing 10,000 cycles at 20 A/g, with only a 3.9% reduction in the capacitance observed; this suggests that the cycling stability was excellent (Figure 7c). The Ragone plots (Figure 7d) showed that the corresponding energy density and power density of symmetric supercapacitors are 6.5 Wh/kg and 125 W/kg, values that are superior to previous reports (Table 1) [20,23,30,31,32]. 

## 3. Experimental Section

### 3.1. Materials

Enzymatic hydrolysis lignin (EHL) was obtained from Shandong Longlive Bio-technology CO., Ltd., (Dezhou, China). The chemicals including KOH, HCl, and polytetrafluoroethylene were bought from Shanghai Macklin Biochemical Co., Ltd., (Shanghai, China). Acetylene black was purchased from Shenzhen Kejingzhida Technology Co., Ltd., (Shezhen, China). Deionized water was used throughout the experiments.

### 3.2. Preparation of LACs

First, 30 g enzymatic hydrolysis lignin (EHL) was fully mixed with a certain amount of KOH. The KOH/EHL mass ratios were R = 2, 3, 4, and 5. The resulting samples were loaded into nickel crucibles, and the nickel crucibles were coated with calcined coke to provide an oxygen-free atmosphere. These were first heated at 550 °C for 2 h and then raised to various activation temperatures T (T = 600, 700, 800, and 900 °C) for 3 h at a heating rate of 5 degrees Celsius per minute. The obtained carbon samples were purified, washed, and dried and named LAC_800-2_, LAC_800-3_, LAC_800-4_, LAC_800-5_, LAC_600-4_, LAC_700-4_, and LAC_900-4_, respectively.

The material characterization and electrochemical measurements are described in detail in the Supporting Information.

## 4. Conclusions

In summary, EHL-derived activated carbons were prepared using a facile one-step KOH activation approach. The results indicated that the microstructure and capacitive performance of the as-prepared LACs were significantly affected by the KOH/EHL ratio and activation temperature. The optimized LAC_800-4_ sample presented an interconnected porous structure with a high surface area of 2285m^2^/g and an appropriate pore size distribution. When assembled into a symmetrical supercapacitor, the LAC_800-4_ electrode exhibited a specific capacitance of 186.8 F/g at 0.5A/g. Furthermore, the LAC_800-4_ symmetric supercapacitor showed excellent cycling stability, with only a 3.9% reduction in capacitance observed when 10,000 cycles were performed at 20 A/g. The LAC_800-4_ symmetric supercapacitor displayed a high energy density of 6.5 Wh/kg in 6 M aqueous KOH when the power density was 125 W/kg.

## Figures and Tables

**Table 1:** Comparison of the electrochemical performance of reported biomass-derived carbon materials.

Raw Materials	Activator	S_BET_ (m^2^/g)	Electrolyte	Capacitance (F/g)	Current Density (A/g)	Power Density (W/kg)	Energy Density (Wh/kg)	Ref.
Tea waste	KOH	1354	6 M KOH	98	1	5870	2.7	[20]
Chitosan	KOH	2787	6 M KOH	188	0.5	250.1	6.53	[23]
Sugarcane bagasse	KOH	1260	1 M H_2_SO_4_	225	1	3600	5	[30]
Banana peel	KOH	2452	1 M Na_2_SO_4_	138	0.5	2690	5.3	[31]
Gardenia	KOH	1637	6 M KOH	153	0.5	246.9	5.2	[32]
enzymatic hydrolysis lignin	KOH	2285	6 M KOH	186	0.5	125	6.5	This work

## Data Availability

Data are contained within the article or Supplementary Materials.

## Supplementary Materials

The following supporting information can be downloaded at https://www.mdpi.com/article/10.3390/molecules30010089/s1, Electrochemical measurements.
