# Correction: Pan et al. Lignin-Derived Activated Carbon as Electrode Material for High-Performance Supercapacitor. *Molecules* 2025, *30*, 89

**DOI:** 10.3390/molecules30030521

**Published:** 2025-01-24

**Authors:** Chenghao Pan, Yongfeng Ji, Suxia Ren, Tingzhou Lei, Lili Dong

**Affiliations:** 1 School of Environmental Science and Engineering, Changzhou University, Changzhou 213164, China; s22030857045@smail.cczu.edu.cn; 2 Institute of Urban & Rural Mining, Changzhou University, Changzhou 213164, China; 13222666378@163.com (Y.J.); rensuxia@cczu.edu.cn (S.R.)

**Adjustment of Authorship Order**

The corresponding authors were listed in an inappropriate order in the original publication [1]. The corrected authorship order and affiliation appears here.

Chenghao Pan ^1,2^, Yongfeng Ji ^2^, Suxia Ren ^2^, Tingzhou Lei ^2,^* and Lili Dong ^2,^*

^1^  School of Environmental Science and Engineering, Changzhou University, Changzhou 213164, China
^2^  Institute of Urban & Rural Mining, Changzhou University, Changzhou 213164, China

* Correspondence: leitingzhou@163.com (T.L.); dongli2050@cczu.edu.cn (L.D.)

The authors state that the scientific conclusions are unaffected. This correction was approved by the Academic Editor. The original publication has also been updated.
