# Supplementary material for: Lignin-Derived Activated Carbon as Electrode Material for High-Performance Supercapacitor
Source: Molecules. 2024 Dec 29;30(1):89. doi: 10.3390/molecules30010089 (PMC11722339; doi:10.3390/molecules30010089)
Supplement: Supplementary file 1 [file molecules-30-00089-s001.zip › molecules-3368983-supplementary.pdf]

Supporting Information

Lignin-Derived Activated Carbon as Electrode Material for High-Performance Supercapacitor

Chenghao Pan [1,2], Yongfeng Ji [2], Suxia Ren [2], Lili Dong [2,*] and Tingzhou Lei [2,*]

Electrochemical measurements

The electrochemical testing was performed on a Princeton VMC-4 electrochemical workstation with a three-electrode system in 6 M KOH. A Hg/HgO electrode was used as a reference electrode, and a platinum foil was used as the counter electrode. The working electrode was prepared as follows. Firstly, $LAC_{T\text{-}R}$ (80wt%), acetylene black (10wt%), and polytetrafluoroethylene (10wt%) were mixed with ethanol to form well blended slurry. Then, the slurry was rolled into a sheet and punched into a disk-like film with 8 mm diameter. Finally, the electrode sheet was pressed onto the surface of the Ni-foam disk with a pressure of 10 MPa and then dried at 70 °C for 24 h.

The specific capacitance (C, F/g) of the working electrode was calculated from the GCD curves using the following equation:

$$C = \frac{I\Delta t}{m\Delta V} \quad \text{(S1)}$$

where I and m represent the current density and active substance mass, Δt and ΔV represent the discharge time and potential window.

The symmetrical two-electrode system was assembled in a 2032-type coin cell with 6 M KOH and tested on CT3002A battery tester (LAND Electronic Electronic, Wuhan). For this two-electrode cell, the specific capacitance (C, F/g) of a single electrode, the energy density (E, Wh/kg) and power density (P, W/kg) was obtained based on the following equation:

$$C = \frac{2I\Delta t}{m\Delta V} \quad \text{(S2)}$$

$$E = \frac{C\Delta V^2}{8*3.6} \quad \text{(S3)}$$

$$P = \frac{3600E}{\Delta t} \quad \text{(S4)}$$

where I (A) is the constant discharging current, Δt (s) is the discharge time, m (g) is the

loading weight of LACs in a single electrode, and ΔV (V) is the voltage change in the discharge process.
